# Retraction: Angry, old, male – and trustworthy? How expressive and person voice characteristics shape listener trust

**DOI:** 10.1371/journal.pone.0229473

**Published:** 2020-02-13

**Authors:** Annett Schirmer, Yen-ju Feng, Antarika Sen, Trevor B. Penney

After this article [1] was published, the authors notified the journal that the voice frequency and intensity analyses forming part of the reported study were done incorrectly in that they did not exclude pauses or unvoiced elements. The authors also subsequently noted small errors in the computation of the F0 trace due to inconsistent voicing (e.g., fry). To address these issues, they revised the acoustic analyses, and also repeated all of the statistical analyses with adjusted modelling approaches. This showed that the results and conclusions reported in the original article were not consistently supported or replicated. Specifically, the results and conclusions depending on voice acoustics were substantially affected by the analysis errors, whereas results and conclusions as regards to speaker affect, age and sex changed minimally.

A member of *PLOS ONE*’s Editorial Board reviewed the reanalyses and advised that the main conclusions of the article are no longer supported.

Given the extent to which the analysis issues impact the results and conclusions of the article, the authors retract this article. The authors plan to publish an updated version of the article that reports the corrected analyses, results, and conclusions.

All authors agreed with retraction.
